# Thinking Beyond Food to Nutrition and Beyond Cells to Immunology

**DOI:** 10.3390/nu17071254

**Published:** 2025-04-03

**Authors:** Renata Ramalho

**Affiliations:** 1 Nutrition Lab, Egas Moniz Center for Interdisciplinary Research, Egas Moniz School of Health and Science, 2829-511 Caparica, Portugal; rramalho@egasmoniz.edu.pt; 2 Nutritional Immunology—Clinical and Experimental Lab (NICE Lab), Clinical Research Unit, Egas Moniz Center for Interdisciplinary Research, Egas Moniz School of Health and Science, 2829-511 Caparica, Portugal

By the time I was tasked to lead a Special Issue on the “Impacts of Micronutrients on Immune System and Inflammatory Diseases”, seeing publications from so many different areas was something I could not have expected. Nutrition as a science has followed a sometimes tortuous path, with greater or lesser acceptance by the other health sciences, with longer roots in society. But humans have always eaten. And they have probably always suspected that food could make them sick, kill them and, surprisingly, strengthen them and even cure them [1]. It would take millions and millions of years of evolution, but this empirical knowledge would be recognised as a health science, evidence-based, and would integrate multidisciplinary clinical and research teams all over the world [2,3]. This recognition made it possible to invest in nutrition research beyond the food itself: What is in the food matrix that interferes with metabolic processes [4,5]? What is in pathology that can be modulated by nutrition/nutrients [6,7]? How would nutrition/nutrients affect the response to personalised medicine approaches [8,9]? These questions have driven scientists towards new approaches in clinical nutrition. So, I applaud those who take a step forward, go against the flow and challenge the common/obvious ways of looking at science.

We are at a crucial moment for humanity. These moments require courage. It is vital to acknowledge the necessity of demonstrating fortitude in confronting the inescapable reality that the environment is becoming a primary cause of human illness [10,11,12,13]. Moreover, there is a pressing need to assume responsibility for the aspects of our environment that are within our capacity to modify, and to proactively initiate transformative changes [14]. This can only be achieved when researchers defy themselves and take their avant-garde thinking to societies. Nutritional Immunology has been at the forefront of both nutrition and immunology [15]. It has challenged scientists to think outside the box [16]. It has been a call for nutritionists to expand their knowledge of the immune system, and for immunologists to recognise that nutrition is a huge part of the equation; every immune cell needs to be fed and behaves differently depending on how we feed it [17,18,19]! But this nutritious broth should not only be enjoyed by professionals in these two scientific fields. It was therefore very interesting to see that the studies published in this Special Issue come from authors with different backgrounds and clinical expertise. Furthermore, these contributions surpassed the common diseases we are used to associating with chronic inflammation and overnutrition: obesity, diabetes and cardiovascular disease.

The study from Gonçalves AM et al. [contribution 1] focused on the role of vitamin D in critical care patients with severe SARS-CoV-2 pneumonia. If there is anything to be learned from COVID-19, it is that we should learn as much as we can about how to prevent this infection and how to act promptly on those who are affected by serious complications. Critical illness from COVID-19 pneumonia is associated with acute hypoxic respiratory failure, and mortality remains high in mechanically ventilated patients [20]. In their study, the authors conducted a single-centre, randomised, non-blinded trial involving 207 patients admitted to a polyvalent intensive care unit with severe SARS-CoV-2 pneumonia. Patients were randomised to receive high (500 MU), moderate (3 MU/day) or no cholecalciferol supplementation, and 25vitD levels, LL-37 levels and VDR expression were assessed as primary outcomes. The results of the study indicate a significant difference in 25vitD levels between patients who received higher doses of cholecalciferol and those who did not, on the third and seventh days. These findings suggest that only supplementation with higher doses of cholecalciferol can significantly increase 25vitD levels in patients with severe SARS-CoV-2 pneumonia and improve their prognosis. These results underscore the potential of vitamin D to enhance clinical outcomes in critical care patients, although further research is warranted to substantiate these findings.

The study by Nishi K et al. [contribution 2] has shifted the focus to cellular research. This fundamental research allowed the authors to explore the potential synergistic anti-inflammatory effect of nobiletin, a polymethoxyflavone particularly common in citrus peel, and docosahexaenoic acid (DHA), in lipopolysaccharide (LPS)-stimulated RAW 264.7 cells. These authors used the combination index (CI) to assess the existence of a synergistic, additive or antagonistic effect of nobiletin and DHA on nitric oxide (NO) production by LPS-stimulated RAW 264.7 cells. Both nobiletin and DHA independently showed inhibitory potency in NO production. Interestingly, however, these authors were able to show that simultaneous treatment with both nobiletin and DHA significantly inhibited NO production in a synergistic manner, as the calculated CI values were less than 0.9. These results are particularly interesting because they highlight the importance of in-depth study of the relationships between nutrients in the food matrix, especially when considering their effects on metabolic stress pathways and inflammation. This exciting field could help to explore synergistic anti-inflammatory effects of combined food ingredients. This could lead to the development of novel foods with increased anti-inflammatory activity, with lower doses of nutrients and fewer side effects.

The paper by Rezai T et al. [contribution 3] focused on the mechanisms behind the influence of breast milk on neonatal mucosal immunity. In their review, they examine the potential mechanisms by which cell-free DNA (cfDNA) in breast milk may influence the development of the immune system in the newborn, through the Toll-like receptor 9 (TLR-9) signalling pathway and interactions with the gut microbiota. The importance of investigating how newborns respond to different types of cfDNA is discussed by the authors. A perspective translational approach to future research is explored in this review, from in vivo studies addressing the stability and functionality of cfDNA in breast milk as it traverses the neonatal digestive system to mechanistic, longitudinal and interventional studies, ultimately determining the therapeutic potential of cfDNA in preventing or alleviating inflammatory conditions in neonates, such as necrotising enterocolitis.

Neuroinflammation and neuroimmune metabolism were explored by Guerreiro D et al. [contribution 4]. In their review, the authors gathered evidence on the potential therapeutic role of the ketogenic diet in modulating neurogenic inflammation and neuroimmunometabolism in refractory epilepsy. This difficult-to-treat condition calls for research into non-traditional approaches. In this review, the authors explore the immune components selected by microglia, astrocytes and neurons that link neuroinflammation to epilepsy, present a summary of upregulated signalling pathways in epilepsy that are involved in neuroinflammation, and dissect the potential modulating effect of a ketogenic diet on the Th17/Treg homeostasis disruption characteristic of refractory epilepsy. Different variants of the ketogenic diet and potential adverse effects of the ketogenic diet that should be considered in clinical practice are also discussed.

Data on the molecular aspects of biotin and its associated molecules, particularly in the context of diseases involving acute and chronic inflammation, were compiled by Sakurai-Yageta et al. [contribution 5]. The review also explored the potential therapeutic applications of biotin, offering a comprehensive and systematic examination of the current knowledge in this field. Biotin deficiency has been demonstrated to have a significant impact on the development and function of lymphocytes in lymphoid tissues. Although rare, biotin deficiency is more likely to be present during pregnancy, during total parenteral nutrition without supplementation, in patients undergoing isotretinoin treatment for acne, elderly individuals, individuals with alcohol use disorder, and smokers (particularly women) [21]. In their analysis, the authors of this review proceed further by gathering evidence on biotin-dependent inherited metabolic disorders, the role of biotin in diabetes, allergies, multiple sclerosis and inflammatory bowel disease, and highlighting a scientific gap that needs to be filled.

As I reflect on the contributions to this Special Issue, I have to say that I truly believe that thinking outside the box is something reserved for the brave, and of course for those behind the brave, who nurture them with encouragement and support, never letting them stray from the path. So, the future of personalised medicine/nutrition will certainly be in the hands of brave people.
